# Real-Time Personalized Monitoring to Estimate Occupational Heat Stress in Ambient Assisted Working

**DOI:** 10.3390/s150716956

**Published:** 2015-07-13

**Authors:** Pablo Pancardo, Francisco D. Acosta, José Adán Hernández-Nolasco, Miguel A. Wister, Diego López-de-Ipiña

**Affiliations:** 1 Juarez Autonomous University of Tabasco, Carr. Cunduacan-Jalpa Km. 0.5, C.P. 86690, Cunduacan, Tabasco, Mexico; E-Mails: francisco.acosta@ujat.mx (F.D.A.);adan.hernandez@ujat.mx (J.A.H.-N.); miguel.wister@ujat.mx (M.A.W.); 2 DeustoTech - Deusto Institute of Technology, University of Deusto, Avda Universidades 24, Bilbao 48007, Spain; E-Mail: dipina@deusto.es

**Keywords:** personalized monitoring, ambient assisted working, occupational heat stress, ISO, wearable sensors, ambient intelligence

## Abstract

Ambient Assisted Working (AAW) is a discipline aiming to provide comfort and safety in the workplace through customization and technology. Workers' comfort may be compromised in many labor situations, including those depending on environmental conditions, like extremely hot weather conduces to heat stress. Occupational heat stress (OHS) happens when a worker is in an uninterrupted physical activity and in a hot environment. OHS can produce strain on the body, which leads to discomfort and eventually to heat illness and even death. Related ISO standards contain methods to estimate OHS and to ensure the safety and health of workers, but they are subjective, impersonal, performed *a posteriori* and even invasive. This paper focuses on the design and development of real-time personalized monitoring for a more effective and objective estimation of OHS, taking into account the individual user profile, fusing data from environmental and unobtrusive body sensors. Formulas employed in this work were taken from different domains and joined in the method that we propose. It is based on calculations that enable continuous surveillance of physical activity performance in a comfortable and healthy manner. In this proposal, we found that OHS can be estimated by satisfying the following criteria: objective, personalized, *in situ*, in real time, just in time and in an unobtrusive way. This enables timely notice for workers to make decisions based on objective information to control OHS.

## 1. Introduction

Ambient Assisted Working (AAW) is an emerging area in ambient intelligence [1], where solutions are proposed to address specific worker needs, taking into account environmental and physiological variables with values provided by different kinds of sensors.

Care of workers in AAW has specific challenges that need to be addressed in order to ensure that people work comfortably. A comfort zone has to be defined to identify environmental and physiological variable values for a specific person to work in a comfortable way.

AAW faces the challenge of monitoring workers' health and comfort, maintaining the best environmental conditions, recognizing potentially dangerous events and promptly alerting staff who are responsible in the workplace. AAW can support monitoring and control in so-called heat stress.

Heat stress is a health condition for people performing physical efforts in hot environments without enough resting periods. The heat stress level values may go from discomfort until heat stroke, or even death [2].

Standard methods proposed by ISO estimate “occupational heat stress” based on environment parameters and the level of physical activity of workers, metabolic rate estimation or even invasive physiological measurements of the human body.

Proposed ISO methods are generic (based on standard characteristics of people and common trades) and subjective, since they are supported by visual observation or by the results from questionnaires answered by workers about their daily activities. Although there are precise objective methods based on physiological measurements, these studies are based on invasive laboratory tests where workers are performing programmed activities, extracting them from their “natural” workspace and work conditions.

These methods prevent knowing objectively the heat stress level to which each specific worker is exposed at every moment of their workday. It is necessary to have a system to automatically monitor the heat stress without requiring exhaustive human supervision or artificial testing in a laboratory. This system should operate in the worker's natural environment whilst they perform their daily work activities.

In this paper, we propose an ambient-assisted working process for workers' heat stress estimation and alerting them in a timely manner when the stress condition is going to rise. The process is customizable to the user profile and intensity of their physical activity and in real time. It is based on continuous monitoring using non-intrusive technology, taking into account user physiological and workplace environmental values. Thus, the heat stress condition can be known immediately for each specific worker in order to ensure his comfort working zone during his journey.

The rest of the paper is organized as follows. In Section 2, the related work is given. Heat stress estimation methods in accordance with ISO are explained in Section 3. In Section 4, the proposed heat level estimation process, methods and materials are presented. Results of experiments are shown in Section 5. In Section 6, our results and findings are discussed. Conclusions and perspectives are drawn in Section 7.

## 2. Related Works

Related works can be divided into two groups. The first one is focused on activity recognition, specific movement detection or energy expenditure estimation. In [3], an accelerometer is used to obtain information that indicates whether all work activities are being performed in the right way. Other studies [4,5] show how to detect unusual movements or postures that could damage the health of workers; in [6], a wearable physiological status monitor is used to analyze relationships that might exist between physical stress and productivity. In [7], the goal is activity classification and energy consumption estimation, but based on the number of METs (metabolic equivalent tasks), according to the compendium of activities [8]; therefore, this does not apply to any free activity undertaken. We want to know the number of METs as a personalized measure of physical effort intensity and energy consumption, not derived from a catalog of trades and occupations [8], but from real-time measurements.

The second group is aimed at estimating heat stress in workers, where most of the proposals are based only on environmental parameters [9,10] or predict short-term estimations making use of machine learning [11].

The reviewed studies do not follow our approach. This proposal integrates four essential aspects to more accurately determine the heath stress level (HSL), real-time data from environmental and physiological sensors, *in situ* and the user profile.

Monitoring for AAW has been applied to diverse areas, such as: sports [12,13], the construction industry [4,6], agriculture [10], the mining industry [14], firefighters [15], labor comfort and health [16]. Most of the research is aimed at increasing productivity or automating activities.

## 3. Ambient-Assisted Working and Standards for Heat Stress

Ambient-assisted working-associated steps must be taken to improve the quality of life using ambient intelligence in the workplace. To achieve this, steps must to be made to ensure that workers are protected from any illness, disease or injury due to their employment activities, as established in the principles of the International Labour Organization (ILO), a specialized agency of the United Nations. ILO estimates that 160 million people are suffering from work-related diseases and recommends the introduction of a preventive safety and health culture, the promotion and development of relevant instruments and technical assistance, thus contributing to the health and well-being of workers and strengthening the economic competitiveness of companies [17].

The physical risk of heat stress arises when people work in hot environments, performing physical activities with significant effort without taking enough resting periods. The International Standards Organization (ISO) has standards containing methods to estimate and control OHS. They are used as a basis for local standards in many countries, and they are applied to estimate the OHS.

The ISO 7243 standard takes into account environmental parameters (temperature, relative humidity and wind velocity) and worker's physical activity level. This includes the easiest method to calculate heat stress, and it indicates the percentage of resting time per hour in accordance with the obtained values. ISO 7933 uses environmental parameters and worker's metabolic rate estimation, which is convenient, because stress is a result of external and internal factors; however, its aim is the evaluation of the thermal stress (using a method for predicting the sweat rate) to determine exposure times in which the physiological strain is acceptable, but it requires environmental and physiological parameters, which are difficult to obtain. ISO 9886 considers physiological measurements of the human body to estimate the response to the work. This standard provides greater accuracy, because its results are obtained in specialized laboratories. We show in Table 1 some disadvantages for each ISO standard.

The ISO standards are independent and are applied commonly in an exclusive way. In order to do something more precise, we propose a solution that integrates the benefits of each standard and contributes to compliance.

None of the methods proposed by ISO meet the objectives of being personalized continuous monitoring *in situ*, in real time and unobtrusive. An important step is to know, just in time, how much energy a worker has spent as a result of continuous physical effort.

Technological advances, such as small accelerometers and devices to measure heart rate, make an effective portable, unobtrusive, continuous monitoring *in situ* and real-time solution possible. Some related works have already demonstrated that fusing (combining) sensor data is a good option to estimate energy expenditure [19]. In our case, this is very convenient, because it implies an objective and effective method. The proposal applies to scenarios that are naturally hot (tropical areas) or artificially heated (ovens, asphalt work).

## 4. Heat Stress Level Estimation

**Figure 1:**
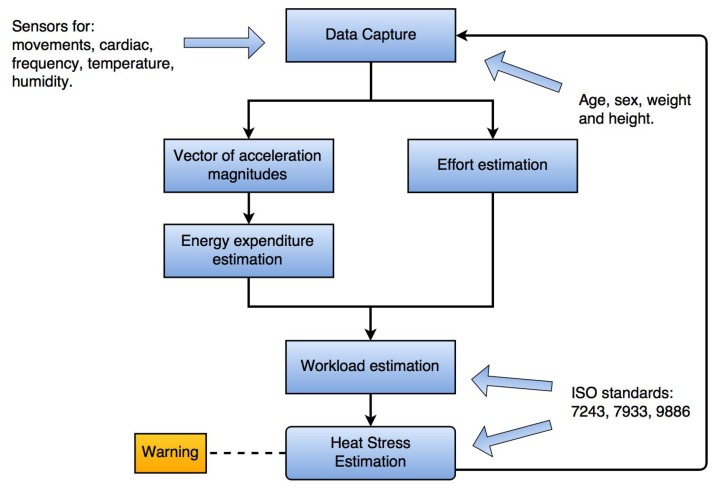
Proposed heat stress estimation method.

The proposed method to determine the HSL is illustrated in Figure 1. We determine the movement intensity with a test device that measures acceleration. Three levels of movement intensity were determined depending on the workload effort: light, moderate and vigorous. We classify effort intensity into seven levels with data obtained from a cardiac frequency sensor, as a second opportunity to know the labor effort for a user.

### 4.1. Data Capture

In this step, instruments (accelerometer) were calibrated, setting their values to those used in the literature, and environmental parameters (temperature and relative humidity) were captured. Temperature and humidity taken from smartphone sensors were homologated to a wet bulb globe thermometer in accordance with the ISO 7243 standard. We used smartphone temperature and humidity sensors because of the portability, low cost and ease of data reading. In order to obtain personalized values, we introduced subject's characteristics (age, sex, weight and height) to feed the estimation algorithms. Cardiac frequency was measured after six minutes of resting and after the user had run 100-m upstairs in a building, to obtain the personal minimum and maximum cardiac frequency values, respectively.

Proposed continuous monitoring is better when all needed sensors are available in the smartphone, but the innocuousness of their sensors to temperature and humidity of the body must be proven. When sensors are not included in the smartphone, values could be obtained from external sensors. Ambient temperature and humidity were obtained at the beginning and at the end of each activity with good results, because values are very close to those obtained with continuous monitoring. In some cases, they can even be calculated from standard formulas, like in the case of cardiac frequency (*maximum cardiac frequency* = 220 − *age*), with lost of personalized results.

### 4.2. Vector of Acceleration Magnitudes and Physical Effort Estimation

Acceleration level and cardiac frequency have a correlation with effort intensity, so smartphones were attached to the user's hip and wrist and fastened with elastic bands. The aim was to chose which of the two was better positioned to represent physical effort for each activity. Values from the *x*, *y* and *z* axes were saved. Sensor data filtering was done using a low-pass technique to attenuate the effect of frequencies from small involuntary movement [20], which does not correspond to a physical activity. We do not use a high pass filter, because we do not need to identify a specific activity.

One of the most popular methods to calculate physical activity intensity is summarizing accelerometer output over the three spatial axes to obtain a scalar value [21]. To estimate the effort in physical activity, we need to sum the vector of acceleration magnitudes (VAM) and determine to what level it corresponds.

Hence, the VAM is calculated according to the following formula:

(1) $$\text{VAM}=\sum_{t_{0}}^{t_{0}+T}(|a_{x}|+|a_{y}|+|a_{z}|)dt$$

where *a_x_*, *a_y_*, *a_z_* are low-pass filtered accelerometer values corresponding to the *x*, *y* and *z* axes. The interval of summation (T) is 60 s (*i.e.*, the epoch, which is a quantitative measure of physical activity in a certain period of time). In previous work [22], it has shown that a period of one minute is relevant.

We record axes values (*x*, *y*, *z*), each 20 ms, which means 50 records per second. Gravity values per minute are obtained from axes values. VAM is used to obtain equivalent METs consumed. This process is detailed in Section 5.1.

In order to obtain effort estimation (worker's physical effort level), we employed a method based on cardiac frequency, because this kind of parameter has a precision accuracy of 90% in effort estimation, in accordance with ISO 9886.

Table 2 shows the Chamoux proposal to know labor activity's absolute cardiac cost (ACC), which is obtained using the average cardiac frequency (ACF) and the resting cardiac frequency (RCF) for a person in each moment. RCF is obtained after a person has slept (8 h) and is resting.

Estimation for ACC is shown in Equation (2).

(2) ACC=ACF−RCF

For example, a person with ACF = 95 (from 70–120 bpm) and an RCF = 70 has an ACC equal to 25.

Chamoux proposed a formula considered personalized. Then, we have to obtain a theoretical maximum cardiac frequency (TMCF) and apply it to Equation (2), as is shown in Equation (3), called the relative cardiac cost (RCC).

(3) $$\text{RCC}=(\frac{\left[\text{ACC}*100\right]}{\left[\text{TMCF}-\text{RCF}\right]})$$

Conventionally, TMCF is obtained from 220 minus the person's age; however, if we consider a real personalized TMCF, then we can request that the user performs an extenuating activity (such as climbing stairs as fast as he/she can), and then, we measure his/her cardiac frequency, so we could obtain a value of 152, for example; then we have:

$$\text{RCC}=(\frac{\left[25*100\right]}{\left[152-70\right]})=30.48$$

where the RCC is a real personalized value.

Table 3 shows effort levels considering the relative cardiac cost for the user. We considered seven levels, going from very light to intense, from cardiac frequency data, in accordance with Chamoux [23], adjusting this criteria to three levels (Table 3) (light, moderate and vigorous) in accordance with ISO 7243. We used cardiac frequency readings from device Zephyr and applied the Chamoux method to know how exhausting each activity is for the workers [23].

We have grouped into three levels the values of relative cardiac cost obtained by the method of Chamoux, in order to favor compliance with ISO Standard 7243. For cost values between zero and 19, the effort is considered light; for costs of 20–39, the effort is moderate, and it is vigorous for values higher than 39.

### 4.3. Energy Expenditure Estimation

We used VAM calculation to translate it to METs. This allowed us to obtain an effective energy expenditure estimation considering physical intensity level in each moment during a workday. A MET is a measure unit of exercise intensity that expresses how many milliliters of oxygen (VO_2_) are consumed per kilogram in a minute. VO_2_ represents the overall metabolic challenge that an exercise imposes. One MET, which is equal to 3.5 mL/kg per minute, is considered to be the average resting energy expenditure of a typical human being. The intensity of exercise can be expressed as multiples of resting energy expenditure.

The metabolic rate or energy consumption was obtained according to physical intensity levels over time. In physical movements, energy expenditure is correlated with acceleration; this means that a higher acceleration in the physical movements involves more energy expenditure. From the sum of the acceleration values for one minute, the METs consumed are mapped according to the correlation established in the calibration phase.

We use the basal metabolic rate (BMR; the energy expenditure quantity necessary to maintain vital functionality) to estimate personal energy expenditure when workers have resting periods during their working day. Conventionally, basal consumption is about 60 percent of daily consumption, so it was pertinent to use a formula that is customized to sex, age, height and weight.

We calculate BMR using the Harris–Benedict equation (based on the personal characteristics of the individual), and map it to METs. The Harris–Benedict Equations (4) and (5) are shown below.

(4) Men BMR=(10*weight in kg)+(6.25*height in cm)−(5*age in years)+5

(5) Women BMR=(10*weight in kg)+(6.25*height in cm)−(5*age in years)−161

### 4.4. Workload Estimation

There are at least two ways to estimate the workload; one based on the intensity of movements related to the activity and another based on cardiac frequency derived from the effort during activity. Movements' intensity is measured in METs, which represents energy consumption, and its value depends on where the movement sensor is placed on the body and the activity undertaken; while does RCC not always have a linear relationship with the intensity of movements (for example, when the activity implies carrying weights).

In our proposal, we first compared VAM values (for each sensor position on the body), and we selected the higher one. The selected value was converted to METs and labeled in accordance with [24] (an energy expenditure inferior to 1.5 METs is labeled as sedentary; from 1.5–3.99 METs is light; between 4 and 6.99 is moderate; and seven or higher is vigorous). Then, the RCC is labeled as the three grouped levels shown in Table 3. Finally, labels for movements and cardiac frequency were compared, and we chose the one that best represents the workload. For example, in the event that an activity (stacking chairs) has a movements intensity labeled as “light” and an RCC labeled as “moderate”, the RCC label is taken, as this best represents the workload. In the case of an activity (sweeping) having a moderate movement intensity and a light RCC, movement intensity is selected, which best represents the workload.

### 4.5. Heat Stress Estimation

To comply with the ISO 7243, the estimation of heat stress levels is considered in this standard, but using workload levels obtained from data captured by our sensors and processed in the mobile device. We also used the environmental values captured by the smartphone's temperature and humidity sensors.

#### 4.5.1. Method for Approximation WBGT

As we said, ISO 7243 recommends an expensive specialized thermometer, called a wet bulb globe thermometer (WBGT); as an inexpensive practical option, we used an approximation formula to WBGT by the Australian Bureau of Meteorology [25], which we call WBGT_*A*_, that only employs thermometers, such as those contained in smartphone, to measure the temperature of the air (environmental) and applies the simplified Equation (6) [26]:

(6) $${\text{WBGT}}_{A}=0.567*T_{a}+0.393*e+3.94$$

where:*T_a_* = Air temperature (°C) and *e* = vapor pressure of water (hPa) (humidity).

The vapor pressure can be calculated from the temperature and relative humidity using Equation (7):

(7) $$e=\frac{rh}{100}*6.105*\exp(\frac{17.27*T_{a}}{237.7+T_{a}})$$

where:*rh* = Relative humidity (%).

The approximation used by the Bureau of Meteorology does not take into account variations in the intensity of solar radiation or of wind speed and assumes a moderately high radiation level in light wind conditions [25].

The use of this approximation may lead to incorrect estimates of thermal stress, particularly in cloudy and windy conditions. Under these conditions, the approximation is likely to lead to an overestimation of the stress. The approximation will also overestimate nighttime and early morning conditions when the Sun is low or below the horizon.

Although the Australian Bureau of Meteorology approximation [25] is not precise under certain conditions, it represents an easy, inexpensive and effective option to obtain the WBGT index, because there is no need for specialized expensive equipment, as considered in the ISO standard.

#### 4.5.2. Heat Stress and Alarms

We used Table 4 (from ISO 7243) as a reference to determine when a worker has heat stress.

From workload and WBGT*_A_*, the system can show each minute to the user if he/she is under OHS. To determine the level of OHS, we take the rating label that implies greater intensity of effort between movement (three levels of workload) and relative cardiac cost (RCC). The scale for the RCC is also grouped into three levels accordingly to ISO. Then, based on the ISO table (Table 4), the person was advised of his stress condition.

Personal estimation of workload levels is going to be the difference between visual appreciation and personalized heat stress estimation. For example, two workers with the same environmental conditions doing the same physical activities may differ in workload level estimation, leading to different results in OHS estimation.

Apart from measuring HSL, the devised solution also includes reactivity features, since it alerts the user when HSL is surpassed and the worker's supervisor in case the recommended HSL is exceeded for a period, ignoring warning messages.

### 4.6. Materials

The device selected was a Samsung Galaxy S4, Android 4.2.2 (Jelly Bean) Operation System, octa-core chipset, 1.6-GHz Quad + 1.2 GHz Quad CPU. Smartphones contained an accelerometer, STMicroelectronics LSM 330, and a Sensirion SHTC1 humidity and temperature sensor.

We also used a GeneActiv accelerometer wristband, a Zephyr Wireless Bluetooth Heart Rate Monitor for Android and Windows Phone 8, an Omron Sphygmomanometer Model HEM-742INT, a Basis B1 Fitness Wristband and a Multifunction Digital Thermometer OBI 292312.

The application to estimate VAM, physical effort and workload was developed with the Java 6.0 language using the ADT tool v22.3.0-887826.

We used a treadmill mark BH Fitness Model Prisma M60 for the calibration phase. It was operated without inclination.

### 4.7. Continuous Monitoring Process

**Figure 2:**
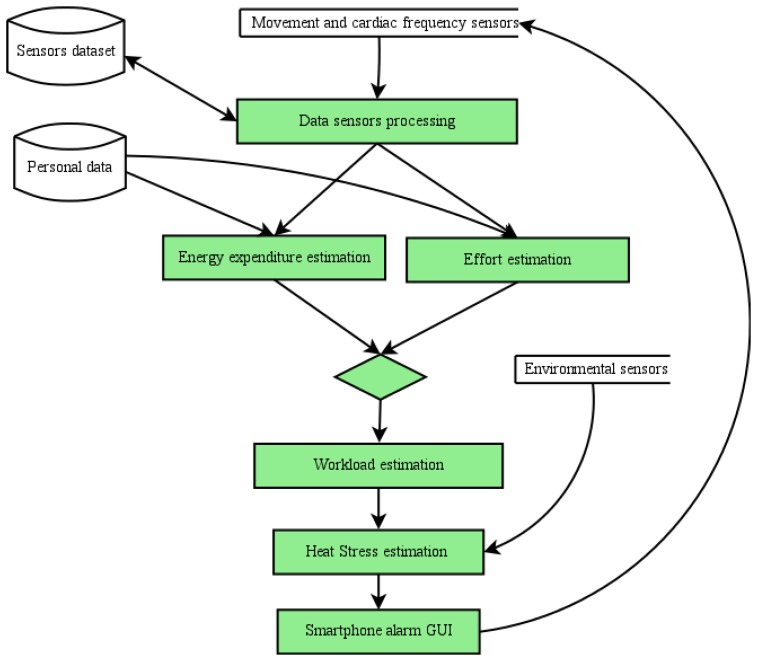
Occupational heat stress (OHS) monitoring process.

The OHS monitoring process of our proposal is shown in Figure 2.

We capture worker's identification and characteristics (age, sex, height and weight) using the GUI prototype. We also captured the value of resting cardiac frequency (RCF) and personal theoretical maximum cardiac frequency (TMCF). Captured data are stored in a database to be recovered when needed. It is demonstrated that accelerometers and cardiac frequency sensors can be used to estimate energy expenditure [27].

Values of the axes (*x*, *y*, *z*) are captured in real time during the execution of physical activities using the smartphone's accelerometer. VAM is obtained from axes values from sensors placed on the hip and wrist using Equation (1). The greatest VAM value per minute is chosen, because it is the best representation of the movements' intensity related to the activity. The chosen value is mapped to METs using Equation (8) obtained in the calibration step of the smartphone's accelerometer, and we map METs to the workload level in accordance with the proposal of [24].

However, there are physical activities having low movement intensity, but not necessarily a lower effort. This is why we propose to use a cardiac frequency sensor to include this kind of activity. We capture cardiac frequency in real time from sensors and apply RCF and TMCF values to Equations (2) and (3), obtaining relative cardiac cost (RCC). The RCC value is used to determine one of the workload levels (light, moderate and vigorous) using Table 3.

After that, we take the workload rating label that best represents physical effort intensity between movements (VAM-METs) and cardiac frequency (RCC).

In order to know whether the worker is under heat stress, we take the smartphone's temperature-humidity sensor values, and we apply Equation (6) to obtain WBGT*_A_*. This smartphone is placed on the wall to avoid the temperature of the worker's body from corrupting the readings of the sensor. We locate in Table 4 the corresponding WBGT*_A_* value and estimated workload to determine the worker condition (estimated OHS). If the worker is under OHS, the corresponding alert message is sent. Later, we restart the process.

## 5. Results

The low-cost proposal is based on a smartphone for continuous monitoring and personalized results. All smartphones are supposed to have a movement sensor. When a frequency cardiac sensor is considered, the precision can be improved, as is stated in this section, but it supposes an extra cost to the solution. With this in mind, the smartphone was placed on the hip or wrist depending on the activity to be measured, even when the simultaneous measurement of both places would give better results, which does not necessarily justify the increased costs (two movement sensors, one on the hip and another on the wrist).

### 5.1. Calibration

Users were dressed in light clothes. This means that clothes are not an influential factor to be considered to calculate the effort.

Calibration participants' characteristics were: age range from 18–46, weight interval 55 kg–81 kg and height interval 1.60–1.70 m.

The Samsung S4 smartphone was placed on the user's hip and fastened with an elastic waistband. Some previous works [28] have demonstrated that the hip is an ideal position to measure physical activity, because it is close to the center of body mass.

**Figure 3:**
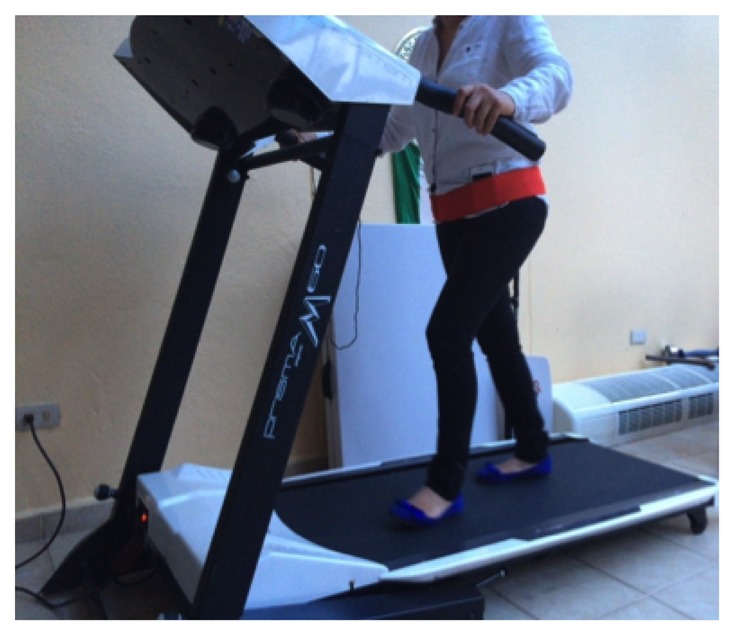
Calibration phase.

Predefined physical activities were walking (3 km/h, 4 km/h and 5 km/h) and running (6 km/h, 7 km/h and 8 km/h). Each user had a resting time of 6 min between each physical activity. The user had had breakfast. We can see the calibration phase in Figure 3.

To analyze the values obtained from the accelerometer during the calibration phase, we used MATLAB Version R2014a.

We processed data with two Infinite Impulse Return digital filters: Butterworth and Type II Chebyshev. We obtained better results with the Chebyshev Type II filter. The Chebyshev Type II is a low pass filter. Data obtained with this filter was compared with those shown in [24], resulting in very similar values. GeneActiv (formerly called Genea) sensors are widely used in scientific research.

In Table 5, we can appreciate the values obtained during the calibration phase.

**Figure 4:**
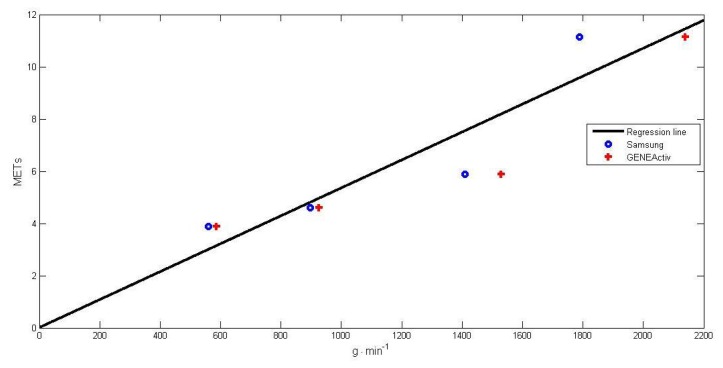
Relationship between GeneActiv and Samsung S4.

A linear relationship was observed when comparing the acceleration values obtained from the smartphone with respect to the published results [21]. Therefore, we estimated the relationship between GeneActiv and the Samsung S4 by means of a mathematical linear regression method. We can see it in Figure 4.

Equation (8) to estimate METs from the Samsung accelerometer data is:

(8) MET=0.005348*VAM+0.01402

where *VAM* = vector of acceleration magnitudes.

For example, when monitoring walking at 5 km/h, the result of the average acceleration obtained was 896.17 g · min^−1^; then, we applied a regression function, and we got a score of 4.80 METs, which is similar to that reported in [21], where indirect calorimetry (oxygen consumption) equipment was used. Therefore, we corroborated the correct correspondence in acceleration (g · min^−1^), between the Samsung's accelerometer and the GeneActiv's accelerometer.

The Zephyr Wireless Bluetooth Heart Rate Monitor was calibrated against two devices, the Basis B1 Fitness Wristband and the Omron Sphygmomanometer, for measuring blood pressure and cardiac frequency. Values obtained with these devices were confirmed by a physician using a stethoscope.

Environmental temperature and relative humidity were taken from the Samsung smartphone sensors, and they were calibrated with external sensors (OBI thermometer) because of its sensitivityto body heat and humidity.

### 5.2. Experiment Results

Participant recruitment was initiated in December 2014 in an effort to obtain a convenience sample of 20 male and female volunteers aged 22–51 years. The recruitment method was as follows: We sent a letter to the custodial staff of the academic department, where the aim of the study was explained and the workers were invited to participate in the testing. We guaranteed the protection of personal data under Mexican law.

Personal characteristics data collection was undertaken from January–May 2015. All participants were free from diagnosed disease and musculoskeletal injury in accordance with their answers. Written informed consent was obtained from each participant. The standards committee of the Informatics and Systems Academic Department of Juarez Autonomous University of Tabasco, Mexico (UJAT for its acronym in Spanish) approved the study.

The experimentation phase was realized with 20 workers (11 men and nine women) who are custodial staff of UJAT. The age range of workers is between 22 and 51 years. They were wearing light clothing; this means that clothing is not a factor that has to be included in the calculation. Otherwise, clothing implications should be considered in the formula. Descriptive characteristics of participants' workers are shown in Table 6.

The period of testing was from March–May 2015, and the type of work evaluated was sweeping smooth floors, glass window cleaning and stacking chairs. Measurements were taken at outdoor workplaces at different times of day to consider *in situ* environmental variables (temperature and humidity). Experiments were performed in the morning (near 26 °C) and at noon (around 36 °C); this is because, in the workplace (tropical area), there is an increase in temperature by up to 10 degrees during the work period. Relative humidity values were near 70%. Therefore, we have different heat stress situations.

**Figure 5:**
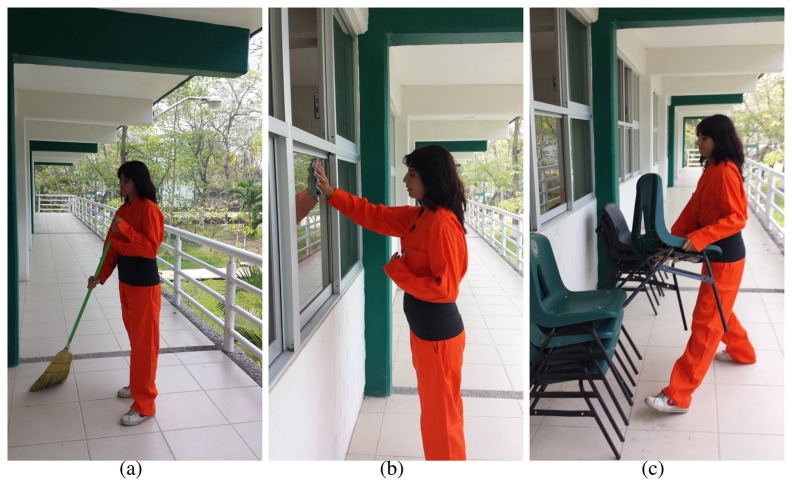
Activities of workers: (**a**) sweeping floor; (**b**) window cleaning; and (**c**) stacking chairs.

Figure 5 illustrates working scenarios corresponding to activities developed by workers in a real labor scenario.

Accelerometer results obtained using the Samsung S4 for three monitored activities are shown in Table 7.

In Table 8, we can see the workers' intensity values of movement on the hip and hand for each activity; values for the sweeping and cleaning glass windows activities reflect a higher intensity of movements than that of stacking chairs; this is because stacking a heavy object implies less intensity of movements, but not necessarily a lower effort. Movement values are taken into account to calculate the amount of METS and workload for each worker.

As shown in Table 9, cardiac frequency is a good indicator of the effort involved in a work activity. These values state the level of effort of each worker. We can notice that values corresponding to sweeping and glass cleaning activities are lower than those of stacking chairs. We assume this behavior of cardiac frequency to be due to the object's weight. This is the reason why in defining the final workload value and its corresponding OHS in Table 10, we have taken from Table 8 the values for sweeping and cleaning activities and from Table 9 the values for the stacking chairs activity.

As we can appreciate in Table 10, workers with OHS (yes) are out of their comfort zone. We can notice that Worker 1 was within his comfort zone when stacking chairs, and he was on the threshold of OHS when cleaning glass windows; this means that he is leaving his comfort zone. Worker 2 is in the same situation for the sweeping activity.

#### 5.2.1. Heat Stress and Alarms

**Figure 6:**
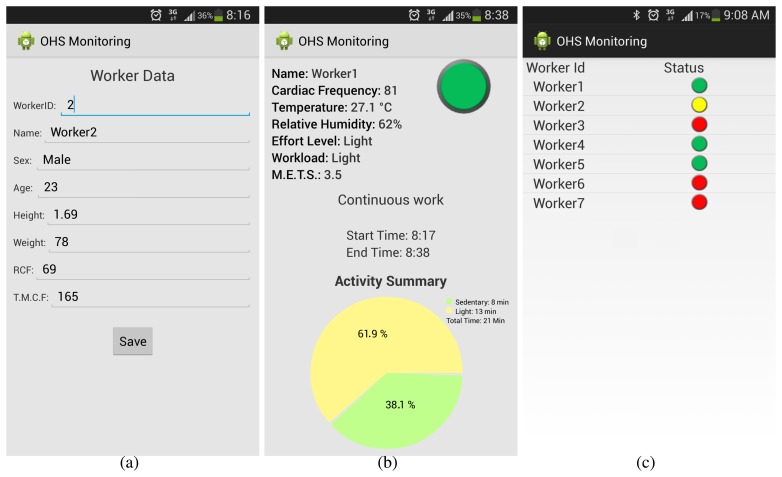
(**a**) Worker data capture; (**b**) worker activity information; and (**c**) supervisor checklist.

We can observe in Figure 6 different GUI screens for the system prototype. Figure 6a shows the personal data capture screen needed in the OHS personal calculation. This screen is intended to appear on the supervisor's smartphone. In Figure 6b, we can see the GUI screen with the user and ambient information, the calculated user effort level, the workload and METS, as well as a user message recommendation over his activity status (the user can continue working in this case) and a summary of activity data. The screen in Figure 6c is intended for the supervisor, offering a general view of the worker's OHS status, helping him to make decisions on special attention when needed.

**Figure 7:**
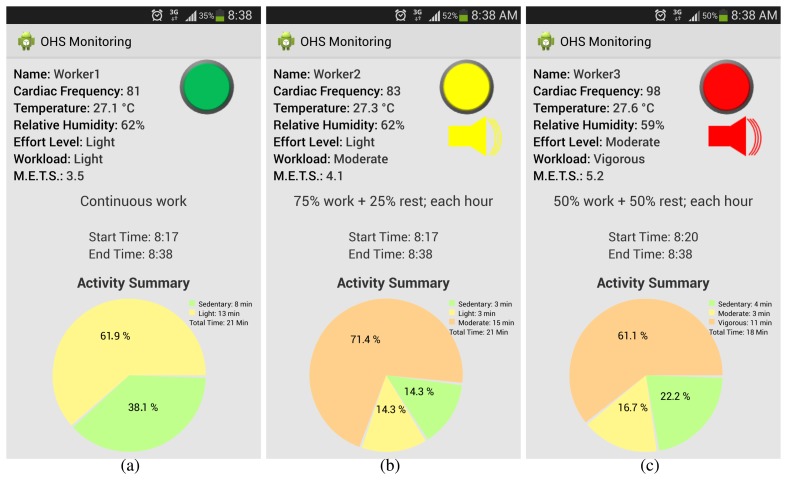
Different OHS levels during morning monitoring: (**a**) without OHS; (**b**) warning alarm previous to OHS; and (**c**) alarm for OHS.

In Figure 7, we can appreciate different OHS values corresponding to monitoring in the morning. The green light means that the user is in his comfort zone; a yellow light corresponds to the user being in the threshold of his comfort zone; and the red one shows a user suffering OHS. The messages' recommendation for each user corresponding to the ISO values workload is shown in Table 4.

**Figure 8:**
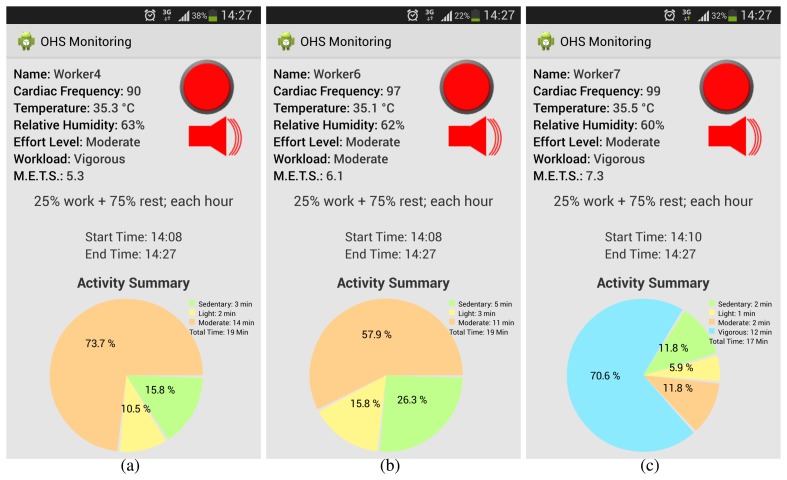
Estimated OHS levels in the afternoon: (**a**) OHS for Worker 4; (**b**) OHS for Worker 6; and (**c**) OHS for Worker 7.

Figures 7 and 8 show the impact of ambient temperature and relative humidity in OHS calculation, because of the ISO compliance.

In Figure 8, we can appreciate different OHS statuses corresponding to monitoring in the afternoon.

## 6. Discussion

The objective of this project was to achieve monitoring for an individualized OHS estimation. We are not trying to reach generalized proposals based on average values of previous studies and profiles of people type, as proposed in the standards, but the input values for the method and the resulting values are for one specific person working *in situ* in normal climate conditions.

This work is in the ambient-assisted working domain, because of the interaction between the worker and the supervisor with the proposed solution. The solution informs the worker and the supervisor who are not necessarily in the same place about the OHS status, taking values of temperature and relative humidity from the environment. The solution has distributed sensors, which are placed on each worker; the solution in ubiquitous, because it is present in each smartphone and it communicates with the supervisor's smartphone to report workers' activities, and the solution knows the workers' status and environmental conditions.

The calculation to determine how much effort is done is based on the intensity of the activity, not its recognition; further study may include activity recognition in order to determine the activity impact on OHS calculation. The intensity of movements is calculated from the speed changes recorded by the accelerometer. This means that in a work activity as in this study, the use of the accelerometer is appropriate. However, for works where the activity is rhythmic and consistent (few changes in speed), the precision measurements of effort can be limited.

Motion sensors, placed on hip or right hand wrist, were used in this paper. Depending on the activity to be measured, the smartphone was placed where the highest acceleration magnitudes were obtained, because obviously they have a better characterization of the activities.

However, for a better characterization of any kind of physical activity, it is necessary to determine the data fusion techniques most appropriate for a precise and formal representation of the efforts involved. This includes fusion of data obtained from sensors of the same kind, and it is mandatory when working with different types of sensors (movement, temperature, light, cardiac frequency, *etc.*). Furthermore, in our study, the smartphone has to be fixed tightly to the user's body in order to eliminate undesired movements, for example when placed in the pocket, proper fusion could avoid the necessity to fix the smartphone tightly, because of the filtering of undesirable movements.

The low-cost approach of the proposed solution does not justify the cost of equipment for indirect calorimetry; instead, the values of METS were obtained from a linear regression equation, which was obtained from the calibration of the Samsung S4 smartphone with respect to the GeneActiv accelerometer (which is scientifically accepted). Therefore, the amount of METs is validated.

The limitation of ISO 7243 standard to address a study in real conditions in the tropics, with temperatures above 31 °C, even in conditions of light work, is that the results would reach high OHS values that do not correspond to reality (which affects productivity). New studies should be performed to adapt the ISO table to tropical heat conditions. In fact, previous studies have proven ISO 7243 to be very cautious [29]. Another ISO limitation is how to capture special considerations (sickness, acclimation to work, physical condition, pregnant workers, *etc.*) in the method.

One objective of this proposal was to provide an economical solution basically supported by a smartphone, which a worker commonly possesses. However, in occupations where primarily hand work is done, it is desirable to have a wrist device that can measure movements and wirelessly transmit the results. This would increase the accuracy of measurements, but substantially increase the cost of the solution. In these cases, suitability between economy and precision must be analyzed.

Reading temperature and humidity through smartphones is an advantage, because the values can obtained from the immediate area where each worker is, since we may have different values of temperature and humidity (microclimates) even in the same work area, even tough, when the smartphone is touching the worker body, the temperature and humidity values may be altered. This is why we claim that the better solution is the measurement of these variables from external environmental sensors. The ISO standard based on the wet bulb globe thermometer does not consider its use for specific worker area with manysensing microclimates surrounding the worker. We can use values offered by meteorological services from cloud computing, when environmental sensors (temperature and humidity) are not available.

Based on the experimental results, we observed that there is no direct relationship between movements and cardiac frequency. For example, when we measured sweeping and glass window cleaning, movement magnitudes rose in a similar way; however, the cardiac frequency does not increase in the same proportion. On the other hand, when chairs are stacked, there are low movement magnitudes, less than sweeping and cleaning, but unlike these activities, the cardiac frequency increases significantly. This justifies the appropriateness of cardiac frequency as an indicator of physical exertion.

The results obtained with the smartphone are reliable and competitive compared to those reported in the literature. Moreover, it is a low-cost, *in situ*, non-invasive solution that can be deployed in real working environments.

Indeed, the results obtained from the sensors reflect the intensity and frequency of workers' physical activities. We verified that user relative cardiac cost is a good indicator of effort level. The cardiac frequency monitor is suitable for activities with little movement, but demanding effort involving oxygen consumption.

Security and data privacy is one of the sensitive issues in information systems within the ambient intelligence domain [30], which is due to the information exchange between components. In addition to this, when it comes to personal data relating to health and job performance, the situation may be even more critical. In our proposal, we request written consent from the workers to handle their data and assure compliance with local laws on data protection.

In our opinion, the proposed monitoring represents a convenient balance between social, economic and productive interests of enterprises to ensure work quality and the welfare of their workers.

## 7. Conclusions and Future Works

As we established in our hypothesis, personalized monitoring for estimating heat stress is more effective and less invasive than ISO methods, because it uses unobtrusive sensor technology to capture in real time environmental parameters and effort intensity.

We found that our monitoring proposal can offer to users a clear vision to interpret energy expenditure and the arduousness index in order to estimate occupational heat stress and employ preventions. Some authors have validated the advantages of sensor technology for activity recognition and to estimate energy expenditure in workplaces [7,20].

Our proposal has important advantages. For instance, it is easy to implement, low cost and employs personalized, real-time measurements instead of table values created from a standard person profile. Furthermore, it is universal and is deployable ubiquitously; its basic infrastructure is a mobile phone and it enables self-care and timely alerting of workers for making decisions about OHS control based on objective information. Last, but no less important, it can be used *in situ* for continuously monitoring the workers.

Wearing a smartphone on the hip is suitable for monitoring work activities that involve a balanced use of the body and limbs. For activities with hands, we must use a sensor bracelet to increase precision.

*In situ* experiments have shown that our monitoring proposal is effective to give users enough data to make decisions to establish a work-resting time program in accordance with heat stress estimation or labor intensity.

Our solution is a good example of how off-the-shelf mobile, personal health and sport monitoring devices, sensibly combined, can give place to a new generation of non-intrusive effective mechanisms to monitor health variables, in this case heat stress level. It also shows how context-awareness, personal sensing and mobile computing can enable ambient assisted working.

Further work will consider recent wristbands containing sensors, such as skin temperature and sweating rate, that would offer new opportunities. It would be interesting to log data across time to analyze correlations with future ailments or diseases; even to build a health monitoring prevention system. New technologies and solutions should also be developed to carry out preventive action.

This study highlights the need for ISO standards to consider including technological devices to build personalized methods.

## Figures and Tables

**Table 1:** Related ISO standards' disadvantages.

**Standard**	**Disadvantages**
ISO 7243	Some studies conclude that the application of its results is too cautious. Measurements do not consider variations in the work rate within a work session. Work intensity has to be established as light, moderate or heavy all of the time. It only employs a standard static worker profile.

ISO 7933	It contains two types of methods to estimate the metabolic consumption. For the first type, tables are used and, in the second, physiological measurements. Both types use values from a standard user (male, 1.8 m height, 70 kg weight, *etc.*), so that is not a custom method. The measurements are not in real time. It only considers standard subjects in good health and fit for the work they perform (ISO 7933). There is growing evidence that it has limited validity and is not usable [18].

ISO 9886	The method requires several physiological measurements, for example body temperature is one of the input data where a valid source is one of the following: esophageal, rectal, gastrointestinal, oral, eardrum, the auditory canal and urine. This is very invasive and requires taking measurements in a laboratory.

**Table 2:** Absolute cardiac cost (ACC) values for a job.

**From ACC**	**ACC for a Job**
0–9	Very light
10–19	Light
20–29	Moderate
30–39	Heavy
40–49	Very heavy

**Table 3:** Effort levels from relative cardiac cost (RCC)

**From RCC**	**Relative Cardiac Cost for User**	**Grouped RCC**
0–9	Very light	Light
10–19	Light	

20–29	A little moderate	Moderate
30–39	Moderate	

40–49	A little heavy	Vigorous
50–59	Heavy	
60–69	Intense	

**Table 4:** Wet bulb globe thermometer (WBGT) limit temperature (°C) values in accordance with workload.

**Work-Rest Regime**	**Workload**		

	**Light**	**Moderate**	**Vigorous**
Continuous work	30.0	26.7	25.0
75% work + 25% rest; each hour	30.6	28.0	25.9
50% work + 50% rest; each hour	31.4	29.4	27.9
25% work + 75% rest; each hour	32.2	31.1	30.0

**Table 5:** Results from calibration. MET, metabolic equivalent task.

**Device**		**Samsung S4 (g·min^−1^)**		**GeneActiv (g·min^−1^)**	

**Activities**	**METs**	**Mean**	**SD**	**Mean**	**SD**
Walking 3 km/h	3.51	340.62	35.91	395	48
Walking 4 km/h	3.88	559.77	13.52	586	10
Walking 5 km/h	4.59	896.17	26.56	926	7.2
Running 6 km/h	5.88	1409.99	85.05	1,529	294
Running 7 km/h	7.98	1677.71	74.23	1890	185
Running 8 km/h	11.13	1788.42	98.50	2139	148

**Table 6:** Descriptive characteristics of the participants.

**Subject**	**Sex**	**Age**	**Height**	**Weight**	**BMI**
Worker1	Male	23	1.63	78.70	29.62
Worker2	Female	23	1.55	52.80	21.98
Worker3	Female	23	1.61	54.30	20.95
Worker4	Male	22	1.69	70.60	24.72
Worker5	Male	23	1.73	84.60	28.27
Worker6	Male	23	1.69	57.20	20.03
Worker7	Female	24	1.68	54.40	19.27
Worker8	Female	33	1.63	83.00	31.24
Worker9	Female	34	1.62	56.90	21.68
Worker10	Male	25	1.70	82.20	28.44
Worker11	Male	28	1.72	83.80	28.33
Worker12	Male	27	1.74	73.80	24.38
Worker13	Male	24	1.80	109.00	33.64
Worker14	Female	28	1.58	73.30	29.36
Worker15	Male	34	1.79	75.70	23.63
Worker16	Female	36	1.62	63.90	24.35
Worker17	Male	33	1.69	70.50	24.68
Worker18	Female	22	1.62	69.00	26.29
Worker19	Female	36	1.61	78.90	30.44
Worker20	Male	51	1.72	68.40	23.12

**Table 7:** Results obtained using the Samsung S4 accelerometer.

**Worker ID**	**Sweeping a Floor**		**Cleaning Glass Windows**		**Stacking Chairs**	

	**On Hip** g · min^−1^	**On Hand** g · min^−1^	**On Hip** g · min^−1^	**On Hand** g · min^−1^	**On Hip** g · min^−1^	**On Hand** g · min^−1^
Worker 1	100.72	752.89	87.52	966.85	344.31	378.15
Worker 2	185.61	772.69	67.14	1378.65	398.24	326.60
Worker 3	143.17	1018.01	87.52	1429.30	318.38	505.00
Worker 4	137.52	846.95	86.55	1179.88	355.01	440.49
Worker 5	143.68	667.08	109.41	1425.93	320.27	399.75
Worker 6	100.20	1226.44	88.94	1660.77	287.44	383.25
Worker 7	144.90	680.28	63.21	1414.77	343.95	432.67
Worker 8	136.20	1097.07	114.00	593.18	378.5	475.02
Worker 9	131.20	1103.00	97.30	1349.16	408.70	468.77
Worker 10	142.20	1089.27	110.90	1082.41	374.90	427.42
Worker 11	153.30	1390.34	127.90	1276.72	325.50	427.38
Worker 12	171.20	1139.57	150.30	1171.09	443.60	699.49
Worker 13	125.40	644.48	118.60	637.19	386.40	481.55
Worker 14	157.10	1152.40	111.60	917.22	343.00	322.44
Worker 15	149.20	754.28	127.00	840.23	373.80	618.88
Worker 16	126.50	857.95	94.40	1478.76	228.8	296.81
Worker 17	123.50	363.90	106.10	1084.99	248.00	425.73
Worker 18	127.80	455.33	111.60	661.99	253.30	305.07
Worker 19	140.40	1395.46	91.60	1262.46	309.10	435.08
Worker 20	160.80	1290.22	115.90	907.79	353.80	425.48

**Table 8:** Selected higher results and their corresponding workloads.

**Worker ID**	**Sweeping a Floor**		**Cleaning Glass Windows**		**Stacking Chairs**	

	**METs**	**Workload**	**METs**	**Workload**	**Mets**	**Workload**
Worker 1	4.04	Moderate	5.18	Moderate	2.04	Light
Worker 2	4.15	Moderate	7.39	Vigorous	2.14	Light
Worker 3	5.46	Moderate	7.66	Vigorous	2.71	Light
Worker 4	4.54	Moderate	6.32	Moderate	2.37	Light
Worker 5	3.58	Light	7.64	Vigorous	2.15	Light
Worker 6	6.57	Moderate	8.89	Vigorous	2.06	Light
Worker 7	3.65	Light	7.58	Vigorous	2.33	Light
Worker 8	5.88	Moderate	3.19	Light	2.55	Light
Worker 9	5.91	Moderate	7.23	Vigorous	2.52	Light
Worker 10	5.84	Moderate	5.80	Moderate	2.30	Light
Worker 11	7.45	Vigorous	6.84	Moderate	2.30	Light
Worker 12	6.11	Moderate	6.28	Moderate	3.75	Light
Worker 13	3.46	Light	3.42	Light	2.59	Light
Worker 14	6.18	Moderate	4.92	Moderate	1.74	Light
Worker 15	4.05	Moderate	4.51	Moderate	3.32	Light
Worker 16	4.60	Moderate	7.92	Vigorous	1.60	Light
Worker 17	1.96	Light	5.82	Moderate	2.29	Light
Worker 18	2.45	Light	3.55	Light	1.65	Light
Worker 19	7.48	Vigorous	6.77	Moderate	2.34	Light
Worker 20	6.91	Moderate	4.87	Moderate	2.29	Light

**Table 9:** Results from Zephyr.

**Worker ID**	**Sweeping a Floor**		**Cleaning Glass Windows**		**Stacking Chairs**	

	**RCC**	**Effort Level**	**RCC**	**Effort Level**	**RCC**	**Effort Level**
Worker 1	13	Light	12	Light	25	Moderate
Worker 2	16	Light	26	Moderate	41	Vigorous
Worker 3	19	Light	9	Light	27	Moderate
Worker 4	10	Light	8	Light	18	Light
Worker 5	16	Light	6	Light	12	Light
Worker 6	9	Light	16	Light	18	Light
Worker 7	14	Light	10	Light	30	Moderate
Worker 8	18	Light	9	Light	16	Light
Worker 9	2	Light	8	Light	26	Moderate
Worker 10	29	Moderate	6	Light	14	Light
Worker 11	6	Light	16	Light	31	Moderate
Worker 12	8	Light	9	Light	13	Light
Worker 13	7	Light	14	Light	27	Moderate
Worker 14	12	Light	11	Light	19	Light
Worker 15	2	Light	17	Light	31	Moderate
Worker 16	29	Moderate	25	Moderate	43	Vigorous
Worker 17	9	Light	9	Light	16	Light
Worker 18	4	Light	8	Light	21	Moderate
Worker 19	7	Light	11	Light	37	Moderate
Worker 20	8	Light	11	Light	21	Moderate

**Table 10:** Estimated OHS.

**Worker ID**	**Sweeping a Floor**			**Cleaning Glass Windows**			**Stacking Chairs**		

	**Workload**	**WBGT***_A_*	**OHS**	**Workload**	**WBGT***_A_*	**OHS**	**Workload**	**WBGT***_A_*	**OHS**
Worker 1	Moderate	28.01	Yes	Moderate	26.84	No	Moderate	25.81	No
Worker 2	Moderate	26.90	No	Vigorous	27.22	Yes	Vigorous	27.76	Yes
Worker 3	Moderate	28.23	Yes	Vigorous	28.34	Yes	Moderate	28.07	Yes
Worker 4	Moderate	31.08	Yes	Moderate	32.24	Yes	Light	31.72	Yes
Worker 5	Light	35.29	Yes	Vigorous	36.13	Yes	Light	34.86	Yes
Worker 6	Moderate	38.09	Yes	Vigorous	36.47	Yes	Light	37.55	Yes
Worker 7	Light	36.26	Yes	Vigorous	35.50	Yes	Moderate	36.33	Yes
Worker 8	Moderate	35.68	Yes	Light	35.68	Yes	Light	35.77	Yes
Worker 9	Moderate	31.52	Yes	Vigorous	34.32	Yes	Moderate	35.30	Yes
Worker 10	Moderate	35.06	Yes	Moderate	36.44	Yes	Light	35.45	Yes
Worker 11	Vigorous	36.95	Yes	Moderate	35.07	Yes	Moderate	35.65	Yes
Worker 12	Moderate	26.49	No	Moderate	28.76	Yes	Light	27.29	No
Worker 13	Light	33.13	Yes	Light	33.84	Yes	Moderate	34.75	Yes
Worker 14	Moderate	30.73	Yes	Moderate	30.34	Yes	Light	30.41	No
Worker 15	Moderate	32.54	Yes	Moderate	33.50	Yes	Moderate	33.56	Yes
Worker 16	Moderate	30.56	Yes	Vigorous	31.32	Yes	Vigorous	31.51	Yes
Worker 17	Light	32.37	Yes	Moderate	32.96	Yes	Light	33.63	Yes
Worker 18	Light	30.79	Yes	Light	30.30	No	Moderate	29.43	Yes
Worker 19	Vigorous	31.87	Yes	Moderate	31.92	Yes	Moderate	31.94	Yes
Worker 20	Moderate	33.75	Yes	Moderate	34.25	Yes	Moderate	33.39	Yes
